# Author response to: Neoadjuvant chemoradiotherapy or chemotherapy alone for oesophageal cancer: population-based cohort study

**DOI:** 10.1093/bjs/znab154

**Published:** 2021-05-05

**Authors:** S K Kamarajah, S R Markar

**Affiliations:** 1 Department of Upper Gastrointestinal Surgery, Queen Elizabeth Hospital Birmingham, University Hospitals Birmingham NHS Trust, Birmingham, UK; 2 Institute of Cancer and Genomic Sciences, College of Medical and Dental Sciences, University of Birmingham, Birmingham, UK; 3 Department of Surgery & Cancer, Imperial College London, London, UK

*Dear Editor*

We would like to thank Deng *et al.* on their comments and interest in our paper^1^. We have responses to their comments. We agree that treatment effects between both neoadjuvant chemoradiotherapy (nCRT) and chemotherapy should be analysed separately for oesophageal adenocarcinoma (OAC) and squamous cell carcinoma (OSCC). This is because both these subtypes have varying biological behaviours and underlying genotype. For instance, accumulating evidence suggests that OSCC responds better to nCRT, although the exact mechanisms for radiosensitvity remain unclear.

Looking to the future, focus of research should be aimed at identifying newer chemotherapy regimens both in neoadjuvant and adjuvant settings in improving long-term survival for both OAC and OSCC. Firstly, understanding the implications of tumour genotype in responses to different neoadjuvant chemotherapy agents such as MAGIC-like ECF or FLOT-like schemes. This would allow a personalised approach towards treating patients with oesophageal cancers. Secondly, role of immunotherapy would be an exciting avenue to explore in this patient population. A recent landmark study evaluated the role of adjuvant nivolumab, a PD-L1 antagonist, in patients undergoing neoadjuvant chemotherapy and resection for patients with OAC and OSCC. This study demonstrated a significant improvement in long-term survival with adjuvant nivolumab in both oesophageal subtypes. This study also raises questions on the impact of other immunotherapy agents in this setting and will provide improvements in long-term survival in this patient cohort.

*Conflict of interest*: The authors declare no conflicts of interest.
